# Supplementary material for: Behavioral, electrocorticographic and electrocardiologic changes in Colossoma macropomum (Tambaqui) in the effect of cunaniol
Source: PLoS One. 2023 Jun 30;18(6):e0287681. doi: 10.1371/journal.pone.0287681 (PMC10313049; doi:10.1371/journal.pone.0287681)
Supplement: Attachment — Submitted filename: Response to Reviewers.docx [file pone.0287681.s002.docx]

December 26^th^, 2022

## Dear Editor, Dr. Michael Esguerra Alba

## PLOS ONE Fish Physiology

RE:  PLOS ONE: Edits requested on your submission PONE-D-22-30736 - [EMID:e24fde6ca6380c67]

I refer to your letter of December 19th, 2022, with your comments and those of reviewers on our manuscript.

We have added some additional details in our Methods section regarding animal care, use and euthanasia procedures considering the PLOS ONE Humane Endpoints Checklist provided. Please find the revised text highlighted in yellow color in the Manuscript.

We hope that the revised manuscript will be acceptable for consideration for publication in *PLOS ONE* and we thank you in advance for your consideration.

Sincerely yours,

Nilton Muto, Ph.D.

Adjunct Professor

Federal University of Para

Perimetral S/N. Guamá

Phone: +55-91-999082020

E-mail: niltonmuto@ufpa.br
