# Supplementary material for: Behavioral, electrocorticographic and electrocardiologic changes in Colossoma macropomum (Tambaqui) in the effect of cunaniol
Source: PLoS One. 2023 Jun 30;18(6):e0287681. doi: 10.1371/journal.pone.0287681 (PMC10313049; doi:10.1371/journal.pone.0287681)
Supplement: Attachment — Submitted filename: Response to Reviewers.docx [file pone.0287681.s003.docx]

March 24^th^, 2023

## Dear Editor, Dr. Michael Esguerra Alba

## PLOS ONE Fish Physiology

RE:  PLOS ONE: Edits requested on your submission PONE-D-22-30736 - [EMID:e24fde6ca6380c67]

I refer to your letter of February 17th, 2023, with your comments and those of reviewers on our manuscript.

We have corrected the manuscript point by point according to reviewer’s comment in red color and added some additional details in our Figures. Please find the revised text highlighted in green color in the Manuscript.

We hope that the revised manuscript will be acceptable for consideration for publication in *PLOS ONE* and we thank you in advance for your consideration.

Sincerely yours,

Nilton Muto, Ph.D.

Adjunct Professor

Federal University of Para

Perimetral S/N. Guamá

Phone: +55-91-999082020

E-mail: niltonmuto@ufpa.br

Response to Reviewer’s Comments:

Reviewer #1: 

The study of Cunaniol-induced seizures in fish is very interesting and holds great potential. I hope that the feedback provided below will enhance the quality of the manuscript and increase its chances of acceptance and publication.
Kind regards.

Comments:

Page 1, lines 1-2: “Cunaniol: Behavioral, electrophysiological changes in Colossoma macropomum (Tambaqui)”. I recommend modifying the title of the manuscript to make it clearer.

As the reviewer suggested, we modified the title of the manuscript. Line 1.

Page 4, lines 93-94: “In relation to its chemical composition, cunaniol C14H14O2 has as nomenclature: (2R, 3S) -2 - [(Z) -non-1-en-3,5,7-triinyl] oxan-3-ol] following IUPAC rules”. Please enclose the chemical formula of cunaniol in parentheses, and also provide the meaning of the IUPAC acronym (International Union of Pure and Applied Chemistry).

As the reviewer pointed out, we provided the acronym of IUPAC and the chemical composition of cunaniol. Line 94-95.

Page 5, lines 103-104: “… potent action of this extract as a chemoconvulsant agent with changes in brain oscillations in Beta (12 to 28 Hz)”. To make the text clearer, I suggest using ‘changes in Beta brain oscillations (12 to 28 Hz)’.

According to reviewer comments, we corrected “changes in Beta brain oscillations..”

Please correct and standardize the bibliographic references in the text to conform to the journal guidelines for citations (https://journals.plos.org/plosone/s/submission-guidelines#loc-references). For example, in the body of the text, you can cite ‘Costa and colleagues (2006) [corresponding reference number here]…’ (page 5, line 105).

Done.

Page 5, line 116: “… to analyze behavioral, and electrophysiological responses in…”. The comma is not necessary.

Done

Page 7, line 166-169: “… and the same tissues were collected for oxidative stress evaluation; (…) at the end of this experiment, muscle, brain and heart tissues were collected to evaluate oxidative stress after contact…”. The results of the experiments measuring oxidative stress are not included in the manuscript. Therefore, I recommend either removing this information from the text or providing an explanation for its intended publication in a future work.

As the reviewer suggested, we removed the information for oxidative stress evaluation

Pages 7 and 8, lines 161-180: Please improve the presentation of the groups treated in each experiment.

Done.

Page 11, lines 255-261: Please clearly indicate the statistical analysis used in each performed experiment.

Done. Line 258-260

Page 12, line 275: Table 1 is redundant as its information is already detailed in the text.

Page 12, line 277: Replace “electroencephalographic record amplitude” with ‘EEG amplitude’.
Done. Line 277.

Figure 1: In Figures 1A and 1B, there is a correlation between the left and right images (1A = 300 s; 1B = 600 s), hence they should be aligned closely. The images in the center are cutouts of the original tracing of the electrophysiological record. Therefore, it is recommended to place them to the right of the original image, which should have the selected sections clearly marked. In my opinion, Figures 1A and 1B should be arranged in a vertical orientation, with each figure having two columns (similar to Figure 3B), to enhance their visual appeal and make interpretation of the images easier.

As the reviewer suggested, we placed the spectral frequency in the middle.

Page 14, line 317: “The normal ECG of C. macropomum is shown in Figures 317 6 A and B, ...”. I believe that the normal ECG is displayed in Figure 5.

According to reviewer’s comments, we replaced the sentences according to Fig 5 to Fig 6. Line”

315 - 320


Page 15, line 355: “… induced seizure outbreaks, changes in the EEG recordings observed in Figure 8 C were…”. The correct figure is 7C.

Done.

Figures 3 and 7: I suggest redoing the figures based on the comment about figure 1.

Done.

Page 18, line 425: I suggest concluding the discussion with questions and perspectives on the use of this substance in future works.

Done.

Finally, I suggest a thorough review of the text to correct errors and improve coherence.

Reviewer #2:

Comments:

The authors should better describe the toxic substance Cunaniol in the introduction.

Unfortunately, there are still few studies describing Cunaniol and its toxicity. The present study is focusing in elucidate better properties of this Amazonian plant.

The authors do not make it clear, whether the effect of cunaniol on bradycardia is direct or indirect?

As the reviewer suggested, we added the information: “The effect on decreasing or increasing cardiac activity in fish may be related to an indirect action mediated by effects on the central nervous system, which during seizures can activate autonomous mechanisms that may explain these changes.” Line 409 – 412.

Cunaniol is an alcohol and could be blocking ionotropic glutamate receptors , why the authors did not test this possibility?

Our objective was to evaluate the blocking of the anticonvulsant effect with classic drugs (phenobarbital, phenytoin and diazepam). To compare the effects of cunaniol with a glutamatergic agonist.

Previously, we published an in vitro study: “Evaluation of uptake glutamate in cultures of retina cells in the presence of cunaniol”. In that study, the cunaniol was able to block the uptake of glutamate in neuronal cell cultures, confirming that this effect was concentration dependent.
